# Video-assisted thoracoscopic esophagectomy: keynote lecture

**DOI:** 10.1007/s11748-016-0650-3

**Published:** 2016-04-29

**Authors:** Miguel A. Cuesta, Nicole van der Wielen, Jennifer Straatman, Donald L. van der Peet

**Affiliations:** Department of Gastrointestinal Surgery, VU University Medical Center, De Boelelaan 1117, ZH 7F020, 1018 HV Amsterdam, The Netherlands

**Keywords:** Minimally invasive esophagectomy, Thoracoscopy, Prone position, TIME trial

## Abstract

Minimally invasive esophagectomy (MIE) by thoracoscopy after neoadjuvant therapy results in significant short-term advantages such as a lower incidence of pulmonary infections and a better quality of life (QoL) with the same completeness of resection. After 1 year, a better QoL is still observed for MIE in comparison with the open approach, while having the same survival. Seven issues about implementation of MIE for cancer require discussion: (1) choice of the extension of esophageal resection and use of neoadjuvant therapy; (2) reasons to approach the esophageal cancer by MIE; (3) determining the best minimally invasive approach for gastro-esophageal junction cancers; (4) implementation of evidence-based MIE; (5) standardization of the surgical anatomy of the esophagus based on MIE; (6) future lines of research of MIE; and (7) learning process. In the time of imaging-integrated surgery it is clear that the MIE approach should be increasingly implemented in all centers worldwide having an adequate volume of patients and expertise.

## Introduction

In 1991, Dallemagne introduced the right thoracoscopic approach in lateral position for esophageal cancer with total lung block, thereby mimicking the conventional approach [1]. Initial reports showed a high conversion rate to thoracotomy and a high respiratory morbidity. Searching for reduction of the conversion rate and the respiratory infection rate, Cuschieri et al. designed the thoracoscopic approach in prone decubitus position so that a total collapse of the lung was no longer necessary for dissecting the esophagus and thereby possibly reducing the rate of respiratory infections [2].

After a feasibility period, the minimally invasive esophagectomy (MIE) approach by thoracoscopy in prone or lateral position or by transhiatal approach is being widely implemented and increasingly performed all over the world for patients with resectable esophageal cancer (EC) to reduce postoperative respiratory complications and to enhance the quality of life by avoiding a right thoracotomy and laparotomy [3–5]. Other important, recent developments in esophageal surgery concern the systematic use of neoadjuvant treatment, such as the use of chemotherapy (MAGIC trial scheme) or chemoradiotherapy (CROSS scheme) [6, 7]. Neoadjuvant therapy for stages 2 and 3 significantly increases 5-year survival of patients with esophageal cancer in both squamous cell cancer (SCC) as well as adenocarcinomas (Adc).

Currently in discussion in the West is the extension of mediastinal lymphadenectomy. Before, in 1994, the ISDE had defined four types of mediastinal lymphadenectomy in treating esophageal cancer (SCC) according to its extension: the standard, the extended, the total mediastinal and the three-field [8]. The advent of imaging-integrated surgery requires a new look at mediastinal lymphadenectomy.

## Statement about minimally invasive esophagectomy (MIE)

The minimally invasive esophagectomy should entail the same operation as the standard open esophageal resection with the only difference being the approach: thoracoscopy instead of thoracotomy and laparoscopy instead of laparotomy.

Seven issues will be discussed about the implementation of MIE for cancer:

1. Choice of the extension of esophageal resection, and use of neoadjuvant therapy.
2. Reasons to approach the esophageal cancer by MIE.
3. Determining the best minimally invasive approach for gastro-esophageal junction (GEJ) cancers.
4. Implementation of evidence based MIE.
5. Standardization of the surgical anatomy of the esophagus based on MIE.
6. Future lines of research of MIE.
7. Learning process.

## The choice of the extension for MIE esophageal resection and use of neoadjuvant therapy

Based on information gathered in Japan about the frequency of lymph node metastases according to tumor location [9] and the evidence obtained by randomized controlled trials (RCT) such as the HIVEX trial [10], middle and upper esophageal cancers may be approached by a three-stage thoracoscopy with total mediastinal lymphadenectomy (LN) and laparoscopy with cervical anastomosis after neoadjuvant therapy. In cases of lower esophageal and GEJ, Siewert 1 and 2, a three-stage or a two-stage Ivor Lewis operation is performed, by laparoscopy and thoracoscopy with standard LN with intrathoracic anastomosis after neoadjuvant therapy [11]. If there is a suspicion of enlarged lymph nodes by PET CT-scan in the paratracheal area in these distal tumors, mostly Adc, LN of these areas is also added. In high-risk patients with distal or GEJ cancers, the laparoscopic transhiatal approach is an option after neoadjuvant therapy.

## Reasons to approach the esophageal cancer by MIE

There is less operative trauma and consequently less morbidity. Performing a thoracoscopy avoids a thoracotomy. Possibly there are fewer postoperative pulmonary complications, especially if no complete pulmonary block had to be used, as done in thoracoscopy in prone position. In laparoscopic transhiatal dissection, the operation is performed under direct vision and probably with less manipulation and retraction of the mediastinum (heart) and therefore less hemodynamic complications. It will add to a better quality of life and perhaps a better survival [12–17].

All surgical approaches used for open esophagectomy have been implemented for MIE. The transhiatal approach, the three-stage esophageal resection, the Ivor Lewis operation, the thoracoscopy in prone position and the esophageal resection facilitated by robot [1–5, 18–21].

## Determining the best minimally invasive approach for gastro-esophageal junction (GEJ) cancers

Gastroesophageal junction adenocarcinomas account for 30–40 % of all esophageal cancers in the West. We use—accepting its limitations—the Siewert classification to locate these tumors leading to implications for the type of neoadjuvant therapy but also for the surgical approach. Many oncologists will indicate neoadjuvant chemotherapy for these tumors. Type 1 is located mainly on the side of the esophagus, type 3 on the side of the gastric cardias and type 2 between both. For type 3 we performed a laparoscopic total gastrectomy [11]. For type 2, an MIE Ivor Lewis procedure is the main choice or the laparoscopic total gastrectomy with an esophagogastrostomy using the Orvil^®^ or a linear stapler anastomosis through the transhiatal approach. Some surgeons will indicate a laparoscopic transhiatal esophageal resection with gastric conduit anastomosis in cervical area and in the case of extensive growth of the tumor along the lesser curvature an open esophageal and gastric resection followed by a colon interposition. Finally for the type 1 tumor, a laparoscopic 2 stage Ivor Lewis or a 3-stage MacKeown approach will be the choice.

The Ivor Lewis approach with intrathoracic anastomosis is a perfect operation for many infracarinal esophageal cancers [22, 23]. Whilst textbook, it is an operation with a high difficulty grade because of the intrathoracic anastomosis. The operation commences with laparoscopy (celiac trunk lymphadenectomy, gastric dissection, creation of a gastric conduit and hiatal dissection) followed by right thoracoscopy (esophageal resection and lymphadenectomy) and intrathoracic anastomosis through thoracoscopy. While there are different types of intrathoracic anastomosis, nonetheless no evidence posits one as graded superior to the other.

In overview, we have the manual anastomosis or an end-to-side anastomosis with a conventional circular stapler (21, 25 or 28 mm after a pursestring suture on the esophageal stump or a prepared Orvil device^®^). Furthermore, the side-to-side anastomosis can be performed using a linear stapler, closing the anterior defect by a transversal suture using conventional suture material or the prepared V-Lock^®^ [24]. Finally the robot-assisted anastomosis is increasingly used permitting a manual high anastomosis in the apex of the thorax because of the ergonomy obtained by the robot [25].

In the Netherlands, anastomotic leaks after MIE Ivor Lewis had initially been reported as high as 14 %, subsequently reduced to current rates holding between 5 and 10 % with a low mortality of 2.1 %. Surgeons must adhere to a proper algorithm for treating these postoperative anastomotic leaks as early as possible, thereby following the maxim that: “Patients who do not progress every day should be studied immediately by CT-scan and endoscopy for assessment of the anastomosis”.

## Implementation of evidence based MIE

There is noteworthy implementation of MIE all over the world. In 2015, using the PubMed^®^ we located 748 papers on MIE esophagectomy and 478 for specifically thoracoscopic esophagectomy. There are four meta-analyses and one randomized controlled trial, being the TIME trial, which compared the total MIE by thoracoscopy in prone and laparoscopy versus the total open approach [12, 26–30]. The French MIRO hybrid trial comparing laparoscopy and thoracotomy with intrathoracic anastomosis versus open approach has already been presented at congresses but has not yet been published [31].

The three most important large series are: the Hulscher’s series with open transthoracic approach in lateral decubitus (114 patients), the Luketich’s series published in 2003 with patients operated by thoracoscopy in lateral position (222 patients), and the Palanivelu’s series of 130 patients operated in thoracoscopic prone position. Comparing these, we see an overall survival rate at 3 years of 40, 34 and 42 %, respectively. Moreover, the comparative rates of pulmonary complications were 57, 20 and 2.3 %, respectively; while the comparative rates of median Intensive Care stay were 6 days, 1 day and 1 day, respectively; and a hospital stay of 19, 7 and 8 days, respectively [3, 4, 10].

These striking differences called for evidence-based analysis of effectiveness. Therefore, from 2010 to 2012 the TIME trial was performed in our department. This was a multicentre, open-label randomized controlled trial [30] comparing thoracoscopy in prone position plus laparoscopy versus right posterolateral thoracotomy and laparotomy followed by intrathoracic or cervical anastomosis after neoadjuvant therapy. Primary end point of the trial was determining the rates of respiratory infections in the first 2 weeks and in-hospital stay, while the secondary end points were the quality of the specimen and quality of life (QoL).We analyzed hospital stay, operative data, postoperative data, complication rate, mortality rates and survival rates.

Concerning the primary outcome, a statistical difference in incidence of postoperative pulmonary infections at end of 2 weeks compared to in-hospital stay was 9 versus 29 % and 12 versus 34 %, respectively, in favor of the MIE group. Concerning the secondary outcomes, hospital stay was statistically different (11 and 14 days) in favor of the MIE; but also different answers in the QoL questionnaires (the SF-36 physical component), EORTC C30 (global health) and OES 18 (taking and pain) were found at 2 weeks after operation in favor of MIE. Moreover other outcomes such as the total of retrieved lymph nodes, the rate of R0 resection (98 and 90 %), and the in-hospital mortality rates (3.4 and 1.8 %) were not statistically different between the two groups. Other outcomes, such as operative time, were shorter in the open group whereas blood loss and the VAS score were less in MIE group. Importantly, the outcomes of technical complications such as anastomotic leakage and thoracic complications were not different between the groups, whilst the only exception being incidence of vocal cord palsy that showed an initial difference of 2 versus 14 % in favor of the MIE group. Explanation for this outcome is difficult but has to be sought in the leakage of CO_2_ from the thorax in the cervical area needed to create a better plane for dissection. The rates of reoperations (14 and 10 %, respectively) were no different between the two groups. Moreover, at 1-year follow up there were no differences in overall and disease-free survival rates between the two groups (around 75 %) yet the QoL questionnaires point out some differences at the 1-year juncture. The global health, the pain and the physical component of the SF-36 were still statistically different after 1 year in favor of the MIE intervention. Explanation for this is obtained by the advantage of avoidance of the thoracotomy with prevention of the postthoracotomy syndrome [32].

Furthermore, there are four different MIE approaches: (a) the lateral thoracoscopic position, the prone position and the semiprone position [1, 2, 33]; (b) the Hybrid MIE type 1 in which a laparoscopy is combined with a right thoracotomy as in the French MIRO trial [31], (c) the Hybrid MIE 2 that combines a thoracoscopy and the laparotomy [13], and (d) the robot-assisted thoracoscopy with standard laparoscopy [21].

Concerning the semiprone position as proposed in Japan, this seems an important addition to the standard prone approach. This includes the possibility to balance the patient from prone to right semi lateral in order to better visualize the supracarinal area and do a better lymphadenectomy along both recurrent laryngeal nerves [33]. Concerning the thoracoscopy in prone approach there are some differences in the position of trocars between Japanese and western world surgeons. The first positions the trocars anteriorly of the scapula adding mostly a small thoracotomy for retraction, whereas the second positions the trocars posteriorly, between the scapula and the spine, adding only a small thoracotomy at the end of the procedure for retrieval of the specimen and introduction of the circular stapler in the case of Ivor Lewis operation.

Differences between the lateral and prone position show that, while in the prone position there is no necessity for selective intubation in the case of cervical anastomosis (we use an insufflation of 7–8 mm Hg CO_2_ for helping retraction of the lung) in the lateral position selective intubation is usually used. When questions arise whether a quick conversion to thoracotomy is needed because of bleeding, sufficient experience assures that conversion may be performed in both positions.

Differences between prone and lateral position are studied by Kubo et al. with two cohorts of 28 patients in lateral and 30 in prone position. Blood loss and duration of systemic inflammatory response were significantly better in the prone group, with a tendency of the respiratory complications to be also lower in the prone group. Their conclusion was that while thoracoscopy in lateral position was safe and feasible, the prone position might be a potentially less invasive procedure than the lateral position [34].

The FREGAT French group compared the 30-day postoperative mortality (POM) between two important cohorts of patients (663 MIE and 2346 open esophagectomy patients) of the French register. Thirty-day postoperative mortality was 3.3 versus 5.7 %, the in-hospital mortality 5.6 versus 8.1 % and at 90-day mortality 6.9 and 10 %, respectively; where the 30-day POM was significantly favoring the MIE. This study suggests that POM is significantly reduced after MIE for EC. This is highly valuable evidence for aiding in decision-making regarding an optimal (hybrid 1 MIE) approach [35].

Concerning long-term survival, Burdall et al. reported three large series in the UK: the open approach (83 patients), the MIE (64 patients) and the hybrid type 1 (187 patients). They found in the long term that the probability of the length of survival for the three groups, even with no adjustment for T or N stage, was greatest for the MIE group [36].

## Standardization of the surgical anatomy of the esophagus based on MIE

The information gathered by MIE has permitted us to describe the concept of the meso-esophagus in the subcarinal area of the thoracic esophagus. During thoracoscopy in prone position we have observed that all structures at subcarinal level, vessels, lymph vessels and nerves are coming from the side of the thoracic aorta to the esophagus. There is a double fascia between aorta and the esophagus that has to be divided in order to perform a systematic step-by-step MIE. In the supracarinal esophagus it is different because the vessels, nerves and lymph vessels are coming on both sides of the esophagus. Description of which structures have to be divided and which to be preserved at this level is important for an adequate esophageal resection and lymphadenectomy with preservation of the arterial vascularization of the trachea and bronchi and proper innervation to the lungs [37, 38].

## Future lines of research of MIE

There are some ongoing RCT’s such as the ROBOT trial that compares the open esophagectomy with the thoracoscopic approach assisted by the robot [21], but also RCT’s that will compare the MIE with the open and hybrid approaches. In the UK and in Japan surgeons still harbor doubts about the advantages of MIE, therefore three new trials have been started. In the UK, the ROMIO trial with three arms: the MIE, the hybrid 1 and the open [39]. In Japan surgeons are comparing the prone position with the open esophagectomy and in China they are going to initiate a RCT comparing the lateral MIE with the open approach [40].

## Learning process

To initiate teaching of the MIE approach, surgeons of a designated proctored Upper GI group will need to have access to an adequate volume of patients with EC and have gained enough experience in open esophagectomy and minimally invasive surgery. Moreover, with the approval of the direction of the hospital and the department they have to organize a dedicated team (at least with 2 surgeons) and visit a center of excellence to learn how this type of intervention has to be performed. Consequently, apprentice learners, will under the guidance of an authorized mentor need to be monitored while carrying out several MIE procedures in their own hospital.

## Conclusion

Minimally invasive esophagectomy after neoadjuvant therapy results in significant advantages on the short term such as lower incidence of pulmonary infections and a better short-term QoL with the same completeness of resection. After 1 year there is still a better QoL with the same survival rate. Three more RCT’s are planned to follow up on the established practice of video-assisted thoracoscopic esophagectomy; together with the publication of the French MIRO hybrid trial, we may expect significant improvements in reducing morbidity and increasing the benefits of the intrathoracic anastomosis.

In the time of imaging-integrated surgery it is clear that the MIE approach should be increasingly implemented in all centers worldwide having an adequate volume of patients and expertise.
